# Magnetic targeted near-infrared II PA/MR imaging guided photothermal therapy to trigger cancer immunotherapy

**DOI:** 10.7150/thno.43604

**Published:** 2020-04-06

**Authors:** Qinrui Fu, Zhi Li, Jiamin Ye, Zhong Li, Fengfu Fu, Syue-Liang Lin, Cheng Allen Chang, Huanghao Yang, Jibin Song

**Affiliations:** 1 MOE Key Laboratory for Analytical Science of Food Safety and Biology, College of Chemistry, Fuzhou University, Fuzhou 350108, P. R. China.; 2 Department of Biomedical Imaging and Radiological Sciences, National Yang-Ming University, Taipei 122, Taiwan (ROC)

**Keywords:** second near-infrared window, photoacoustic imaging, magnetic targeting, photothermal therapy, immunotherapy

## Abstract

**Rationale**: Photothermal therapy (PTT) alone is easy to cause cancer recurrence and fail to completely resist metastasis, yet recurrence and metastasis are two major difficulties in cancer treatment. Titanium disulfide (TiS_2_) nanosheet anchored iron oxide nanoparticles (IO NPs) with strong absorption in the second near-infrared (NIR-II) window and excellent magnetic properties is developed as therapeutic agent for NIR-II photoacoustic (PA) imaging and magnetic resonance (MR) imaging guided NIR-II PTT triggered immunotherapy.

**Methods**: The TiS_2_ nanosheets were prepared through a modified colloidal chemistry approach, and TSIO nanoagents were prepared by using a one pot self-assembly technique. The magnetic targeting capability of TSIO nanoagents were monitored by NIR-II PA, MR and thermal imaging *in vivo*. The NIR-II PTT combined with immunotherapy effect was investigated in mouse breast cancer tumor-bearing mice.

**Results**: The TSIO nanoplatform showed enhanced tumor accumulation when a magnetic field was applied and had the ability to real time monitor the treatment process *via* dual NIR-II PA and MR imaging. In addition, the magnetic targeted NIR-II PA/MR imaging guided PTT provides an effective way to reverse the immunosuppression inside a tumor and to cooperate with immunotherapy to improve therapeutic outcome of the primary, distal and metastatic tumors.

## Introduction

Photoacoustic (PA) imaging, as a noninvasive molecular imaging technology, employs contrast agents that have accumulated within a tumor to generate PA signals with high imaging resolution and contrast, low dissipation and scattering, excellent penetrability and sensitivity, and promises immense potential in biomedical and clinical applications 1-3. Over recent years, the principal focus of PA imaging has been within the first near-infrared window (NIR-I, 650-950 nm). However, the signal to noise ratio and depth of tissue penetration are extremely low because of the high degree of scattering and absorption due to biological tissues and molecules within it, such as skin, lipids, deoxyhemoglobin and oxyhemoglobin in the NIR-I window. Moreover, the main components of biological tissue exhibit strong absorption in the NIR-I window and a decreasing trend within the second near-infrared (NIR-II, 1000-1350 nm) window 4, 5. Very recently, PA imaging in the NIR-II window has received considerable attention due to its enhanced spatial resolution, higher signal to background noise ratio and deeper tissue penetration compared with that in the NIR-I window 6-9. So far, active PA contrast agents in the NIR-II window have been limited to noble metals 10, 11, phosphorus phthalocyanine 12, perfluorocarbon nanodroplets 13, copper sulfide nanoparticles (NPs) 14 and semiconducting polymer NPs 6, 7. However, shortcomings due to potential metal ion leakage, limited retention time in tumors and clearance from the body, poor biocompatibility and biodegradability and unknown long-term toxicity hinder its clinical development. Two-dimensional (2D) materials are promising candidates in the field of theranostics such as bioimaging and photothermal therapy (PTT), due to their broad NIR absorption properties, excellent photothermal performance and extended circulation time 15.

PTT relies on the photothermal effect of photothermal agents that can harvest and convert light energy into heat to raise the temperature of the surrounding environment to cause cancer cell death. PTT has received considerable attention from researchers of cancer therapy because of its negligible invasiveness and considerable satisfactory therapeutic outcome 16, 17. Similar to PA imaging, previous research in PTT has been concerned with the NIR-I window. However, the NIR-II window is more practical because of more effective tissue penetration and higher maximum permissible laser exposure (MPE) for achieving efficient PTT for deep-seated tissue tumors 18. In recent years, PTT in the NIR-II window has attracted increased attention 19-26. Although PA imaging and PTT in the NIR-II window have been developing rapidly, they have done so in isolation, with very few reports of the application of NIR-II PA imaging guided NIR-II PTT due to the lack of suitable nanoagents and advanced PA imaging equipment applicable for the NIR-II window. Therefore, exploration of NIR-II nanoagents with good optical properties and biological characteristics is a promising and meaningful focus of research.

As a classical form of 2D nanomaterial, transition metal dichalcogenides (TMDs) have attracted considerable attention and showed great potential in numerous fields of biomedicine 27-30. Titanium disulfide (TiS_2_) is a classic TMDs material with excellent stability, electrical conductivity and strong absorption in the NIR window, having previously been reported as being used in the detection of biomolecules, as battery electrodes 31, 32 and stamp-transferrable electrodes 33. In our study, NIR-II PA imaging guided NIR-II PTT with TiS_2_ was explored, attributing to its absorption peak can be redshifted to 1000-1350 nm by adjusting the thickness and width of the TiS_2_ nanosheets due to localized surface plasmon resonance 34.

In general, nanoagents rely on the enhanced permeability and retention (EPR) effect to become concentrated in tumors, although the efficiency of this is relative low 35 and largely hinders their further application in biomedical fields. Magnetic targeting could induce magnetic nanoagents circulating in the blood to be directed to the appropriate tumor region with the aid of an applied magnetic field 36. Unlike the EPR effect, magnetic targeting derives from physical interactions and is not restricted by specific receptor expression, which may be the optimal method of tumor targeting. In recent years, magnetic targeted therapy has been widely used in tumor ablation, such as PTT and photodynamic therapy in early stage clinical trials with patients 37-40.

The single PTT method usually does not completely ablate the tumor and is ineffective in the prevention of cancer recurrence and metastasis, yet, metastasis and recurrence are two major difficulties in cancer treatment. The metastasis and recurrence of cancer not only mean that it needs to be treated again, but also can give a fatal blow to the weak body, and even lead to death. Therefore, it is an important task to avoid cancer recurrence and metastasis for improving the survival rate of cancer patients. Recently, cancer immunotherapy has been successful in clinical practice, but this treatment can only benefit a small number of cancer patients 41, 42. One reason is that antitumor infiltrating lymphocytes may be limited by a variety of immunosuppressive mechanisms, such as the inhibition of regulatory T cells (Treg) and the inhibition of the induction expression of the checkpoint receptor 43. In addition, cancer cells with high mutation rate can produce new distinct antigens, and dendritic cells are deficient in the identification of these antigens, thereby evading an immune response 44 .

**Scheme 1:**
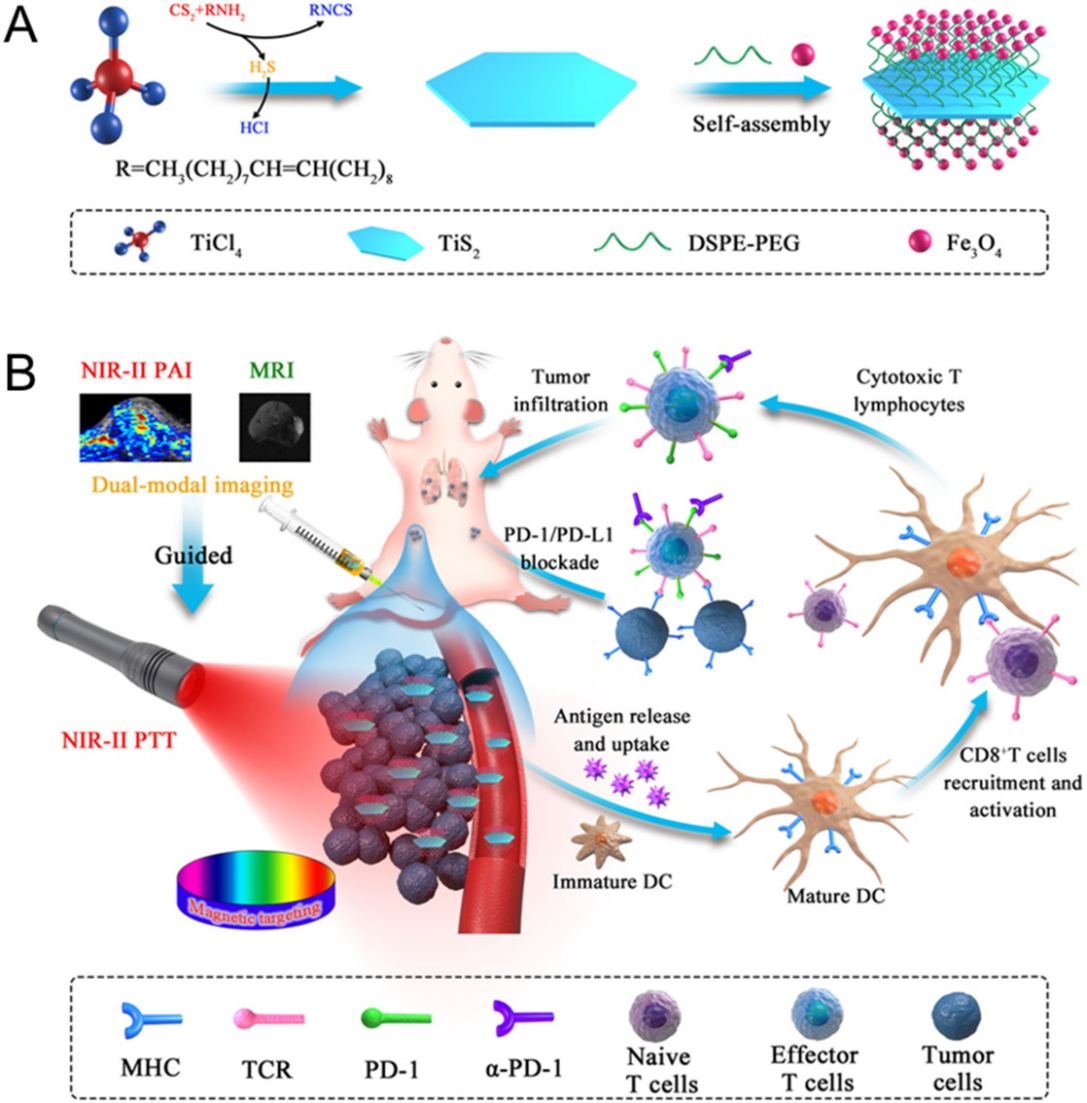
** (A)** Fabrication procedure using a facile one pot self-assembly technique for TSIO nanoagents. **(B)** Schematic illustration of the functionality of TSIO nanoagents that NIR-II PA/MR imaging monitored tumor magnetic targeted accumulation and subsequent NIR-II PTT and PD-1/PD- L1 checkpoint blockade therapy.

Herein, we developed multifunctional 2D TiS_2_-based NIR-II nanoagents for magnetic targeted dual-modal NIR-II PA/MR imaging guided synergistic photothermal-immune combination therapy. Iron oxide (IO) NPs were successfully anchored on the surface of TiS_2_ nanosheets, which was not only an effective method of improving the affinity of the nanoagents to cancer cells and prolonging the residence time using the magnetic targeting effect under an applied magnetic field, but could also be used as a *T*_2_-weighted contrast agent in MR imaging. Nanoagents (termed as TSIO) modified with polyethylene glycol PEG demonstrated excellent biocompatibility and stability in addition to strong absorbance in the NIR-II window, (**Scheme 1**). TSIO nanoagents were effective magnetic vectors that achieved high tumor accumulation under an applied magnetic field, with significantly increased PA/MR signal intensity and therapeutic outcomes. Specifically, the accumulation of nanoagents at a tumor site treated by magnetic targeting reached 17.9% of injected dose (ID/g), approximately 2.5-fold greater than that observed in non-magnetic targeting (7.15% ID/g). Additionally, PA signal intensity at 24 h post-injection in the magnetic targeting group was approximately 1.58-fold higher than that of the group without magnetic targeting. Meanwhile, the systemic anticancer immunity triggered by the TSIO nanoplatform under NIR-II laser irradiation was exploited. The cancer related antigens were generated by post-PTT triggered immunogenic cell death in the presence of immune response amplifier imiquimod. It caused intense anticancer immune responses, which together with PD-1 antibody (*α*-PD-1) could effectively blockade the PD-1/PD-L1 pathway to inhibit mouse breast cancer (4T1) recurrence and metastases by an enhanced abscopal effect. This suggests that TMDs have great development potential in the field of PTT induced immunotherapy and PA imaging in the NIR-II window.

## Results and Discussion

### Synthesis and characterization of IO NP modified TiS_2_ (TSIO)

**Figure 1:**
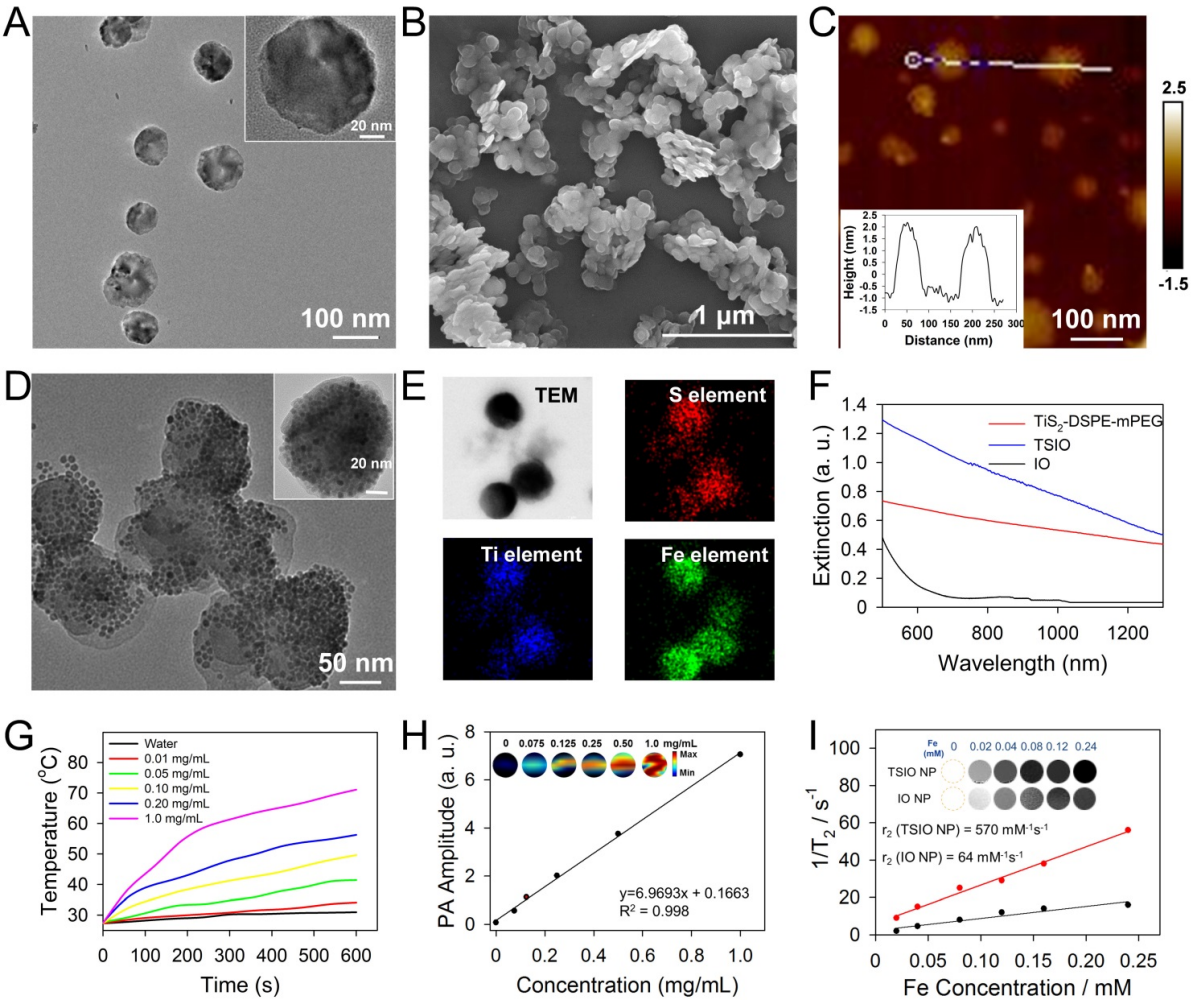
** (A)** TEM **(B)** SEM **(C)** AFM images of TiS_2_ nanosheets and the AFM-measured thickness of TiS_2_ nanosheets (inset of c). **(D)** TEM image and **(E)** EDS elemental maps of TSIO. **(F)** UV-*vis*-NIR spectra of IO, DSPE-mPEG-modified TiS_2_ and TSIO (1.0 mg·mL^-1^). **(G)** Temperature variation of different concentration of TSIO under 1064 nm NIR laser irradiation. **(H)** *In vitro* PA amplitudes and the corresponding PA images of TSIO aqueous solution as a function of concentration (0, 0.075, 0.125, 0.25, 0.50 and 1.0 mg·mL^-1^) at 1270 nm. **(I)** *In vitro T*_2_-weighted MR imaging photographs and corresponding *T*_2_ relaxation rate of TSIO (red line) and IO NPs (black line) in aqueous dispersion at different Fe concentration.

TiS_2_ nanosheets were synthesized through a modified colloidal chemistry approach based on previously reported methodology 34, 45. Briefly, titanium tetrachloride and carbon disulfide, as titanium and sulfur precursors, were injected in turn into a mixed solvent of octadecen (ODE, protecting agent) and oleylamine (OA, growth solvent) with inert gas protection, then heated to 300 °C for 3 h (Scheme 1A). As revealed by transmission electron microscopy (TEM) (**Figure 1**A), the as-fabricated TiS_2_ nanosheet exhibited a hexagonal-like morphology with dimensions in the range 60-100 nm. Scanning electron microscopy (SEM) revealed TiS_2_ nanosheets with 2D sheet structures (Figure 1B), consistent with those in previous reports 34, 45. The mean thickness of the TiS_2_ nanosheets, as determined by atomic force microscopy (AFM), was approximately 3 nm (Figure 1C). The OA-coated TiS_2_ nanosheets were modified using DSPE-mPEG *via* van der Waals force to improve their aqueous solubility and biocompatibility, their morphology tending to become round, as demonstrated by TEM (Figure S1). IO NPs were prepared *via* a classical thermodecomposition strategy 46, having a spherical structure with a typical diameter of 5-10 nm (Figure S2 and S3). The IO NPs were anchored to the TiS_2_ nanosheets through hydrophobic-hydrophobic interactions using a one pot self-assembly technique. As revealed by TEM (Figure 1D), surfaces of the TiS_2_ nanosheets were populated by a large number of IO NPs (Figure S4). Elemental mapping of titanium, sulfur and iron further demonstrated that IO NPs were anchored on the surface of the TiS_2_ nanosheets (Figure 1E). In addition, the nanoagents showed excellent stability in physiological conditions (Figure S5).

### Enhanced photothermal, photoacoustic and magnetic resonance performance of the TSIO nanoagents

To investigate the photothermal performance of the TSIO nanoagents, aqueous dispersions of PEGylated TiS_2_ (TiS_2_@DSPE-mPEG) and TSIO were characterized by UV-*vis*-NIR spectroscopy. There was strong absorbance at wavelengths ranging from 500-1350 nm for both TiS_2_@DSPE-mPEG and TSIO, which encompassed both NIR-I and NIR-II windows (Figure 1F). Subsequently, the photothermal performance of TSIO was further explored through measurement of the temperature of 1.0 mL aliquots of various concentrations of nanoagents in aqueous dispersions after irradiation with 808 nm (NIR-I window) and 1064 nm (NIR-II window) lasers at a power density of 1.0 W·cm^-2^. The TSIO dispersions exhibited a concentration dependent temperature rise in both the NIR-I (Figure S6) and NIR-II windows (Figures 1G). A 1.0 mg·mL^-1^ aqueous suspension of TSIO nanoagents demonstrated a maximum rise in temperature from 27 to 62 °C and 71.1 °C within 5 min and 10 min respectively (Figure S7), with even a low concentration of 200 *μ*g·mL^-1^ achieving a 28.5 °C temperature increment (Figure 1G). To further explore the photostability of the TSIO NPs, a 1.0 mg·mL^-1^ suspension of TSIO was irradiated in cycles with a 1064 nm laser, each cycle consisting of irradiation for 5 min, with subsequent cooling to room temperature. After four cycles, the maximum temperature remained fundamentally the same (Figure S8), the photothermal conversion efficiency (*ƞ*) at 1064 nm was calculated to be 45.51%, and that at 808 nm was 46.82% (Figure S9), revealing that the TSIO nanoagents possessed excellent photothermal performance and stability.

The TSIO had excellent photothermal properties in the NIR-II window and so was deemed suitable as a contrast agent for PA imaging. Firstly, PA spectra of the TSIO NPs were obtained by pulsed laser irradiation ranging from the NIR-I window to the NIR-II window (Figure S10), which were essentially consistent with the UV-*vis*-NIR spectra. Because of its strong signal in the NIR-II window, the potential of TSIO as a NIR-II PA contrast agent was evaluated in an aqueous dispersion at a variety of concentrations. As shown in Figure 1H, PA images of the TSIO NPs gradually became brighter as concentration increased, as did their PA signal intensity as calculated by quantitative analysis, suggesting that concentration dependent PA signal enhancement was achieved.

MR imaging is a powerful and non-invasive clinical diagnostic method. Due to the excellent *T*_2_-weighed MR imaging properties of IO NPs, the TSIO materials, comprising IO NPs could also represent excellent MR imaging contrast agents. The MR image signal intensities of the TSIO dispersions, consistent with the PA imaging results, also exhibited a concentration dependent relationship with signal enhancement (Figure 1I). Much effort has been exerted to increase *T*_2_ contrast efficiency (*r*_2_) so as to improve the quality of MR images. A number of groups, including ours, have speculated that clustering of magnetic NPs might be the most efficient method to enhance *r*_2_ in MR imaging, due to the decreased interparticle distance of clustered NPs that enhanced the magnetic dipole interaction effect 47-50. In this study, we observed that the TSIO NPs exhibited evidently enhanced *r*_2_ signals relative to those of unmodified IO NPs. As shown in Figure 1I, the *r*_2_ values of the TSIO NPs (570 mM^-1^·s^-1^) were approximately 8.9-fold greater than those of IO NPs (64 mM^-1^·s^-1^), which may be attributed to the magnetic-magnetic interaction effect between the tightly deposited IO NPs on the TiS_2_ nanosheets having a shorter interparticle distance.

The results demonstrated that the as-prepared TSIO nanoagents exhibited excellent photothermal and PA performance in the NIR-II window due to their enhanced light absorption properties, in addition to having excellent performance in magnetic resonance, suggesting that the TSIO nanoagents represents a suitable candidate for using as a PTT agent in the NIR-II window against cancer* in vivo*.

### *In vitro* cytotoxic therapy using the TSIO nanoagents

**Figure 2:**
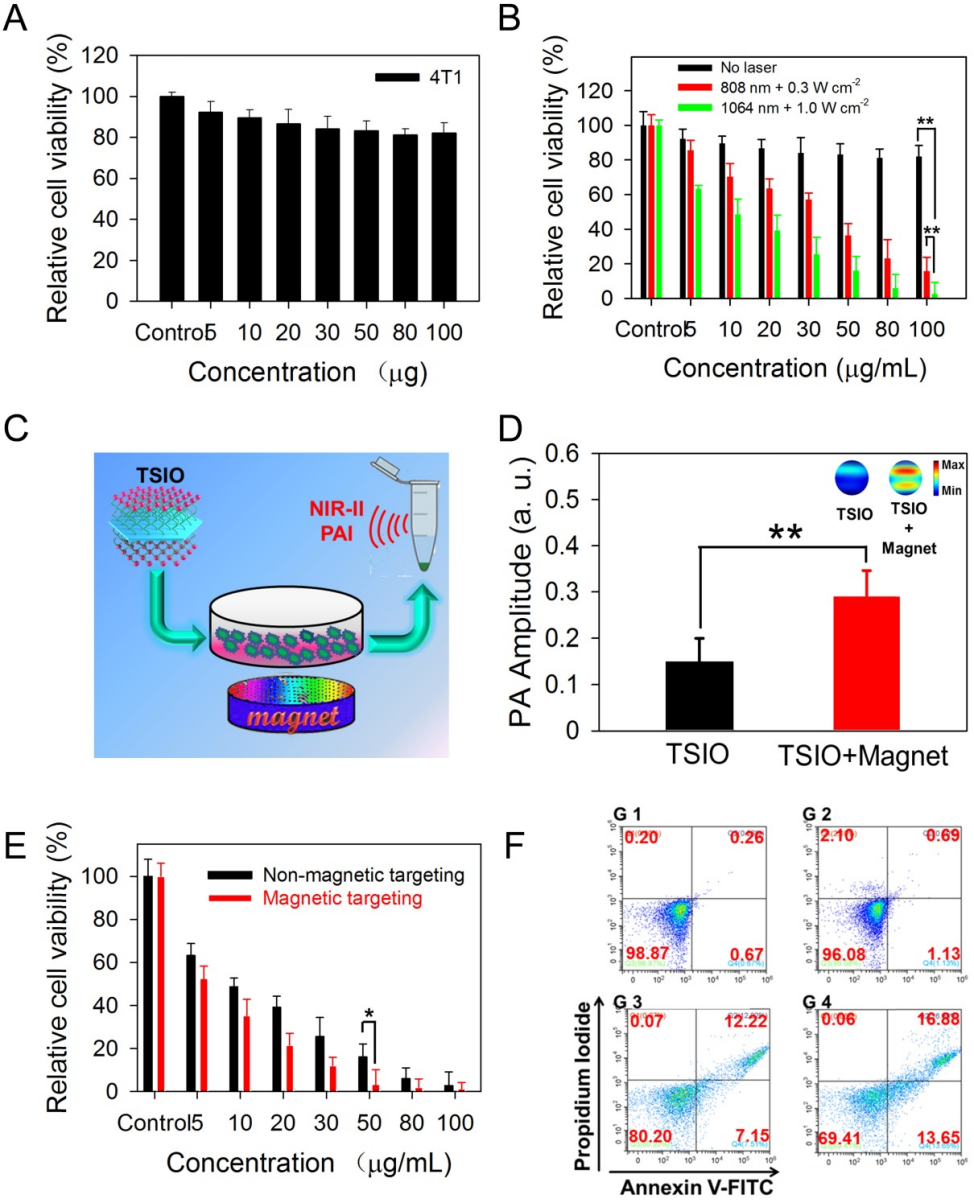
** (A)** Relative cell viability of 4T1 cells treated with different concentration (5-100 *μ*g·mL^-1^) of TSIO for 24 h. **(B)** *In vitro* photothermal efficiency of 4T1 cell treated with TSIO nanoagents under different laser and laser intensities (808 nm with 0.3 W·cm^-2^ and 1064 nm with 1.0 W·cm^-2^) for 5 min. **(C)** Schematic illustration of PA images of 4T1 cell by magnetic targeting treatment. **(D)** PA amplitude of the cells with and without magnetic targeting treatment at 1270 nm. **(E)** The comparison of photothermal efficiency of 4T1 cell treated with TSIO nanoagents with and without magnetic targeting. **(F)** The flow cytometry analyses of 4T1 cancer cells after different treatments (G 1: PBS, G 2: TSIO, G 3: TSIO + NIR-II laser, G 4: TSIO + Magnet + NIR-II laser). (*P* values, *P < 0.05, **P < 0.01).

*In vitro* experiments of cytotoxicity and photothermal performance of TSIO nanoagents were conducted to investigate their potential in biomedical applications. A standard Cell Counting Kit-8 (CCK-8) assay was performed using 4T1 cell lines to explore the cytotoxicity of the TSIO nanoagents. After incubation with the cell lines for 24 h, no apparent biological toxicity was detected, even at a concentration of 100 *μ*g·mL^-1^ of the TSIO nanoagents (**Figure 2**A), demonstrating their relatively good biocompatibility.

Subsequently, the photothermal ablation of 4T1 cells by TSIO at a variety of concentrations was evaluated under NIR laser irradiation. From the skin tolerance threshold (ANSI Z136.1-2007) established by the America National Standards Institute, a 1064 nm laser possess a MPE value of 1.0 W·cm^-2^, while 808 nm laser light has a lower MPE limit of ~0.3 W·cm^-2^ 51, 52. Thus, photothermal performance was studied by irradiating at 808 nm at a power density of 0.3 W·cm^-2^ and at 1064 nm at a power density of 1.0 W·cm^-2^. As shown in Figure 2B, relative cell viability after irradiation with a 1064 nm laser at a power density of 1.0 W·cm^-2^ was significantly lower than with irradiation at 808 nm at a power density of 0.3 W·cm^-2^, demonstrating that irradiation with the higher power 1064 nm laser was more suitable for photothermal ablation of cancer cells.

PA imaging performance is related to the photothermal properties of the samples due to PA signals being generated by broadband ultrasonic waves from transient thermoelastic expansion caused by absorbed light energy 2, 53. *In vitro* NIR-II PA imaging could be employed to monitor the magnetically assisted accumulation of nanoagents in cancer cells (Figure 2C), resulting from the good photothermal performance of TSIO nanoagents in the NIR-II window. PA signals were measured at 1270 nm following treatment of cells with TSIO alone or with magnetic targeting for 12 h. Quantitative analysis demonstrated that the PA amplitude of cells treated with TSIO in combination with magnetic targeting was approximately 1.9-fold greater (Figure 2D), indicating that magnetic targeted uptake of nanoagents by cancer cells. Moreover, the PTT of cells treated using TSIO with magnetic targeting was significantly superior to that of the group without magnetic targeting (Figure 2E). Flow cytometric analysis was further utilized to investigate the effect of magnetic targeting on cancer cells that had ingested nanoagents and PTT. Briefly, after treatment with 50 *μ*g·mL^-1^ TSIO suspension and irradiation with a 1064 nm laser for 10 min, cells were collected for flow cytometric analysis (Figure S11). Cells treated with TSIO combined with magnetic targeting and NIR-II laser illumination exhibited considerable cell apoptosis, approximately 1.55-fold higher than that of the TSIO + NIR-II laser group without an applied magnetic field (Figure 2F), suggesting that magnetic targeting significantly promoted cellular uptake of nanoagents and improved photothermal efficacy.

### Magnetic targeting capability of TSIO nanoagents monitored by multimodal NIR-II PA, MR and thermal imaging

**Figure 3:**
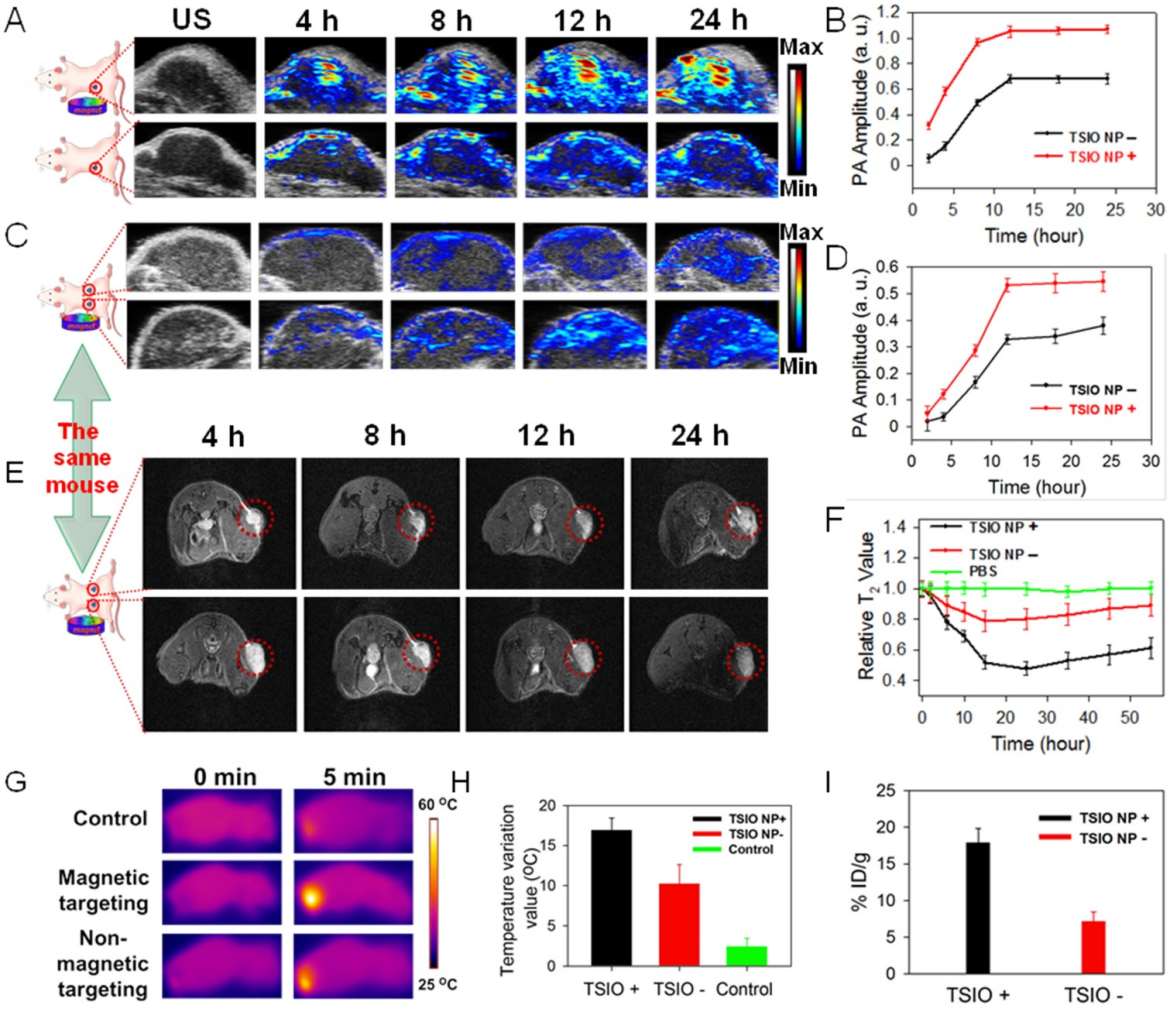
** (A)** *In vivo* NIR-II PA imaging and **(B)** corresponding PA amplitudes of single tumor animal model. **(C)** *In vivo* PA imaging and **(D)** corresponding PA amplitudes of double tumor animal model at different time point post-injection of TSIO nanoagents at 1270 nm. **(E)** *In vivo* MR imaging of tumor-bearing mice with (bottom) and without (top) magnetic targeting and **(F)** corresponding relative *T*_2_ value. **(G)** IR thermal images and **(H)** temperature profile of the mice in control group, non-magnetic targeting group and magnetic targeting group. **(I)** The tumor accumulation efficiency of TSIO nanoagents through using magnetic targeting (TSIO NP+) and non-magnetic targeting (TSIO NP-).

Encouraged by the excellent NIR-II PA signals and superior *in vitro* cell PA imaging performance of the TSIO nanoagents, their capability in *in vivo* PA imaging in the NIR-II window was further evaluated in a single tumor animal model, in addition to their capacity for magnetic targeting and ability to facilitate accumulation in a tumor region. As shown in the **Figure 3**A, the PA amplitude at 1270 nm (NIR-II window) in the tumor site gradually increased over time post-injection of TSIO in both the non-targeted and magnetic targeted groups. In addition, a significant increase in PA intensity was observed within the tumor region 12 h post-injection, remaining almost as great 24 h later, and demonstrating a time dependent accumulation of TSIO nanoagents in the tumor site. Compared with the group that had no magnetic targeting, the PA amplitude of the magnetic targeting group was greater at each time point, especially 24 h post-injection, which was approximately 1.58-fold higher (Figure 3B), confirming again that magnetic targeting promoted uptake of nanoagents by tumor cells.

Due to uncontrollable factors such as individual differences and variations in procedure, the experimental results of a single tumor animal model tend to have large variation. In order to minimize these deviations and improve the precision of the imaging results, a double tumor animal model was established, into which an intravenous injection of 0.2 mL of 1 mg·mL^-1^ TSIO nanoagents were administered (magnetic targeting of one tumor and no targeting of the other). The mice were then imaged using conditions identical to those of the single tumor model (Figure 3C). The increasing trend of PA signal was similar to that observed in the single tumor model, with the PA amplitude of the tumor after magnetic targeted treatment being much higher than that of non-targeted treatment after the same duration (Figure 3D). To assess whether the *in vitro* MR imaging capability of the nanoagents would be replicated *in vivo,* MR imaging of the double tumor animal model was conducted and the magnetic targeting mediated accumulation of nanoagents further investigated. As shown in Figure 3E, a time dependent contrast enhancement of MR imaging in the tumor region was observed post-intravenous injection of the TSIO nanoagents, in both the group without magnetic targeting and the magnetic targeting group. In addition, *T*_2_-weighed MR imaging contrast of the magnetic targeting group was higher than that of the non-targeted group (Figure 3F), further validating the effective accumulation of the TSIO nanoagents within the tumor through the magnetic targeting effect.

The *in vivo* magnetic targeted imaging guided photothermal performance of TSIO nanoagents was evaluated by an infrared thermal camera with monitoring of change in temperature of the tumor region. Infrared thermography demonstrated that the temperature of the tumor area of the magnetic targeted treatment group increased rapidly from 33.1 °C to approximately 50 °C when irradiated for 5 min with a 1064 nm laser (1 W·cm^-2^) 12 hours after the intravenous injection of TSIO nanoagents, which induced sufficient local hyperthermia to ablate the tumor cells. By contrast, the temperature of tumor region without magnetic targeting increased by approximately 12 °C, which was 5 °C less than that of the magnetic targeted treatment group. In control animals, the temperature of the tumor region injected with phosphate buffered saline (PBS) increased by only ~2.5 °C (Figures 3G-H), indicating that the tumor with magnetic targeted treatment exhibited considerably superior PTT performance than without magnetic targeting. Specifically, accumulation of nanoagents at the tumor site treated by magnetic targeting 24 h post-injection reached 17.9% ID/g and 17.63 ID/g, approximately 2.5 and 2.6-fold greater than the group without magnetic targeting (7.15% ID/g and 6.68 ID/g), based on the quantity of Fe and Ti element observed, respectively, as tested by inductively coupled plasma mass spectrometry (ICP-MS, Figure 3I, S12), verifying that magnetic targeting is a promising technique to enhance the therapeutic effects *in vivo* by accumulation of NPs within the tumor site. Furthermore, high levels of Fe element were detected in reticuloendothelial systems such as spleen and liver (Figure S13), due to the size of TSIO nanoagents were much larger than renal clearance cut off.

### *In vivo* magnetic targeted photothermal therapy in the NIR-II window combined with immunotherapy to prevent primary tumor recurrence

**Figure 4:**
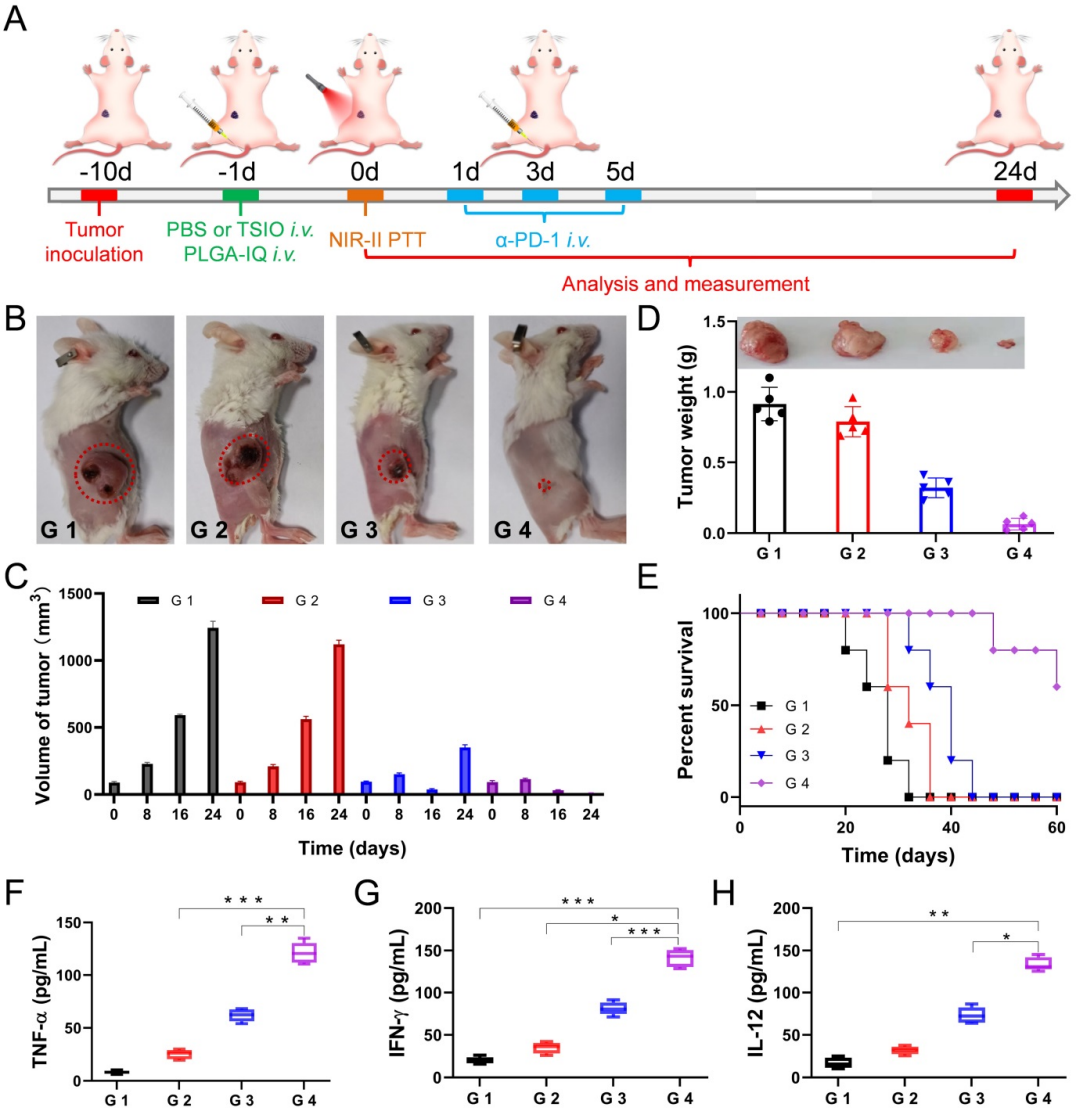
**(A)** Schematic illustration of experimental design for primary tumor therapy. Representative photographs **(B)** and volume **(C)** of and tumor weight **(D)** of primary tumors. **(E)** Survival percentages of the mice in different treatment groups (n=5): G1 (PBS +1064 nm laser), G 2 (PBS + 1064 nm laser + *α*-PD-1), G 3 (TSIO + magnet + 1064 nm laser) and G 4 (TSIO + magnet + 1064 nm laser + *α*-PD-1). The contents of **(F)** TNF-α, **(G)** IFN-γ and **(H)** IL-12. ( *P* values, *P < 0.05, **P < 0.01, ***P < 0.001)

From the experimental stage to clinical application, suitable nanoagents are expected to simultaneously provide effective treatment, therapeutic guidance and monitoring of treatment.^23^ On this basis, the TSIO which has excellent absorption in the NIR-II window, MR imaging ability and magnetic properties could be expected to serve as promising nanoagents for magnetic targeted NIR-II PA and MR imaging guided PTT. However, a single PTT modality usually could not completely cure the cancer and often causes tumor recurrence or metastasis. In recent years, the immune response after PTT has also attracted extensive attention. After the destruction of cancer cells induced by PTT, tumor associated antigens may be released, which triggers a certain level of antitumor immune response. Meanwhile, the combination of PTT and checkpoint blockade therapy could effectively cure the tumor and prevent tumor recurrence and metastasis 54-58. Therefore, we wonder whether PTT with TSIO nanoplatform combined PD-1/PD-L1 checkpoint blockade therapy, would be able to induce tumor specific immune response to prevent tumor recurrence. Thus, the 4T1 tumor-bearing BALB/c mice with a low immunogenicity were used to verify the validity of this hypothesis. 4T1 tumor-bearing mice were randomly divided into four groups (n = 5 per group). A toll-like receptor 7 agonist imiquimod (IQ) was encapsulated by a PEG-PLGA micellar nanoparticle (PLGA-IQ), which was used as an immune response amplifier (the Figure S14 showed that the treatment effect using both PLGA-IQ and *α*-PD-1 was better than that using PLGA-IQ or *α*-PD-1 alone). The schedule of treatment was shown in **Figure 4**A. Briefly, when the primary tumors reached approximately 60 mm^3^, PBS or TSIO nanoagents and PLGA-IQ were injected intravenously and an applied magnetic field was employed to promote high tumor accumulation of NPs, 1064 nm laser irradiation for 5 min was used to the mice of different groups on day 1 post injection, subsequently, mice were intravenously injected with *α*-PD-1 at the dose of 15 *µ*g per mouse on day 1, 3 and 5 post 1064 nm laser radiation.

The experimental results showed that PTT combined with immunotherapy had obvious inhibitory effect on the growth of primary tumor. As shown in the Figure 4B and D, neither *α*-PD-1 alone (G 2 group) nor PTT alone (G 3 group) could effectively inhibit tumor growth and recurrence. In particular, the tumor growth rate of the treatment of *α*-PD-1 alone (G 2 group) was close to that of the control group (G 1 group), suggesting that the treatment of *α*-PD-1 alone had a low treatment effect on cancer. In addition, the individual PTT could not effectively prevent the recurrence of the tumor, the primary tumor in the G 3 group (PTT only) almost disappeared after 16 days of treatment, but a recurrence was followed (Figure 4C), indicating that the non-persistent stimulation was not sufficient to activate a sufficient adaptive T-cell response, and therefore the effect of a complete cure of the tumor could not be achieved. Attributed to the synergistic anticancer effect of PTT and immunotherapy, the primary tumor in the G 4 group disappeared after 16 days of treatment and did not recurred and led to a long-term survival rate of 60% (Figure 4C-E), suggesting the mice treated with PTT-immunotherapy significantly suppress the rate of tumor growth.

In addition, in order to better understand the interaction between PTT and immunological system, the serum of mice was harvested at 24 h after the first injection of* α*-PD-1. Pro-inflammatory cytokines of tumor necrosis factor *α* (TNF-*α*, Figure 4F), interferon-*γ* (IFN-*γ*, Figure 4G) and interleukin-12 (IL-12, Figure 4H) were analyzed by enzyme linked immunosorbent assay (ELISA). TNF-*α* and IFN-*γ* were vital symbols of cellular immunity and played an important role in cancer immunotherapy, while IL-12 played critical roles in the activation of innate immunity. The combination of PTT and immunotherapy (G 4 group) exhibited a level of significant improvement of TNF-*α*, IFN-*γ* and IL-12, implying combined immunotherapy could stimulate systemic immune response and effectively inhibit tumor recurrence.

Although TSIO nanoagents had no significant toxicity* in vitro*, its potential *in vivo* toxicity remains an important issue to be addressed. In our experiments, we carefully monitored the behavior of the mice after tumor ablation *via* PTT-immunotherapy by intravenous injection (20 mg/kg) of TSIO. Within 40 days, no noticeable signs of toxic effects such as abnormalities in body weight, activity, eating, drinking, urination, grooming, exploratory behavior were observed. Mice were sacrificed at day 40 for necropsy, and no significant abnormalities were found in the major organs. Then, major organs of mice were sliced and stained by hematoxylin and eosin for histology analysis (Figure S15), uncovering no obvious organ damage or inflammatory lesions.

### Photothermal-immune combination therapy to inhibit the growth of distal tumor

**Figure 5:**
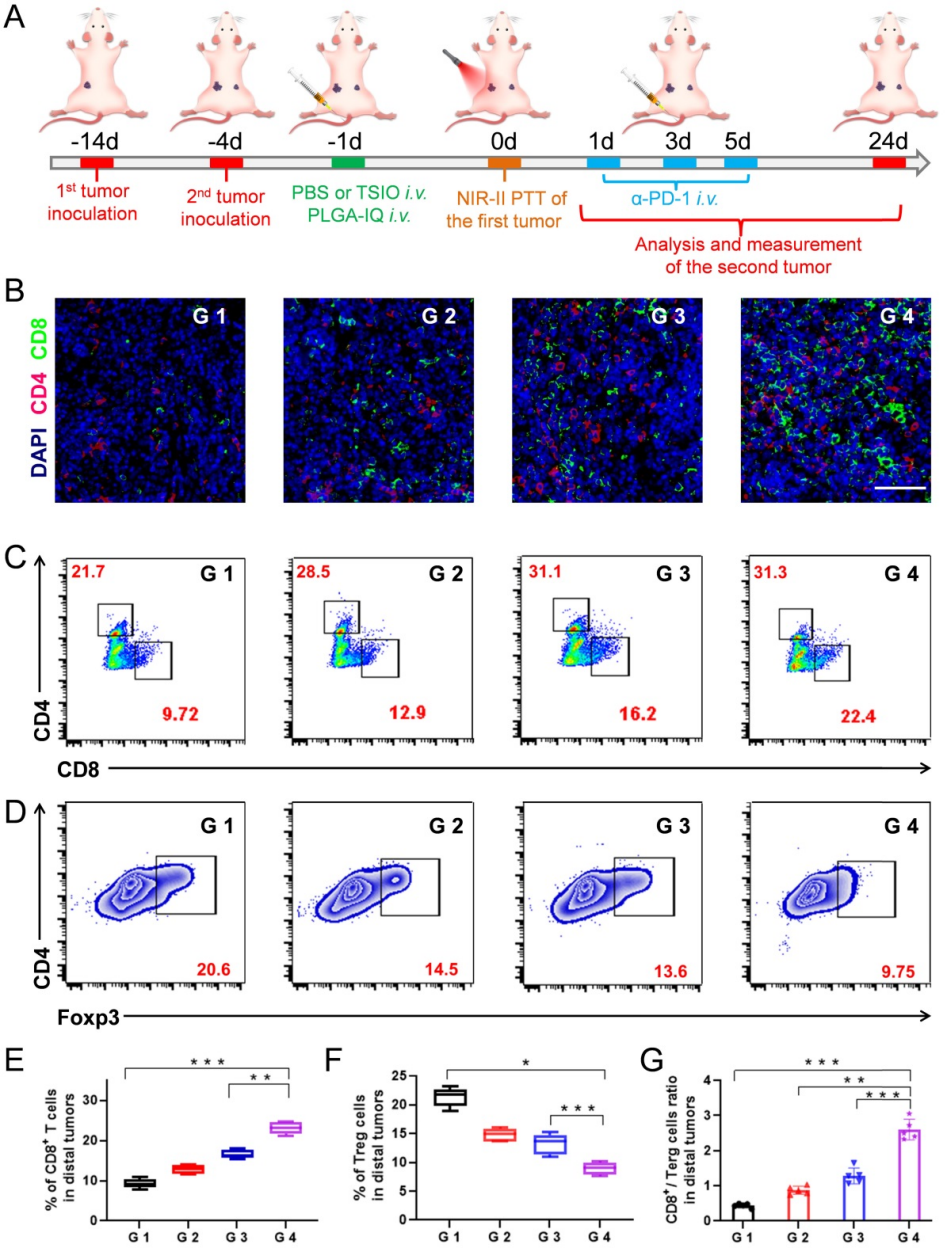
**(A)** Schematic illustration of the experimental design for distal tumor. **(B)** Representative immunofluorescence images of distal tumors showing CD4^+^ and CD8^+^ T cell infiltration (scale bar: 50 µm). **(C)** Representative flow cytometry plots of CD4^+^ and CD8^+^ T cells (gated on CD3^+^ cells) and **(D)** Representative flow cytometry plots of Treg cells in distal tumors. The contents of **(E)** CD8^+^ T cell, **(F)** Terg cells and **(G)** CD8^+^ / Terg ratios in distal tumors (n=5). G1 (PBS +1064 nm laser), G 2 (PBS + 1064 nm laser + *α*-PD-1), G 3 (TSIO + magnet + 1064 nm laser) and G 4 (TSIO + magnet + 1064 nm laser + *α*-PD-1). ( *P* values, *P < 0.05, **P < 0.01, ***P < 0.001)

Encouraged by the ability of photothermal-immune combination therapy to prevent primary tumor recurrence, we then explored its capability to inhibit the growth of the distal tumor. In the study, a bilateral murine tumor model was established (**Figure 5**A). After treatment on primary tumors, the size variations of the distal tumors were measured and recorded (Figure S16). It was worth noting that the growth rate of the distal tumor of the mice injected with *α*-PD-1 after PTT (G 4 group) of the primary tumor was obviously inhibited, and its growth rate of the distal tumor was far lower than that using the *α*-PD-1 (G 2 group) or PTT (G 3 group) alone, which confirmed that the combination of PTT with PD-1/PD-L1 checkpoint blockade therapy played a critical role in systemic immunity establishment and distal tumor suppression.

The mechanisms of anticancer immune responses after combined PTT-immunotherapy with TSIO nanoplatform were investigated. Both CD8+ T cells and CD4+ T cells are T lymphocytes, which play the important roles in the immune response to anticancer therapy. Treg is one of the most important factors to maintain the immune tolerance of the body. Foxp3 is one of the key transcription factors to control the development and function of Treg, and is a key factor to control the expression of immunosuppressive molecules. Therefore, CD8+ T cells, CD4+ T cells and Foxp3 in distal tumors were assayed by immunofluorescence (Figure 5B) and flow cytometry. The combination of PTT with PD-1/PD-L1 checkpoint blockade therapy (G 4 group) led to 2.3-fold more CD8+ T cells and 1.44-fold more CD4+ T cells than that of the control group (G 1 group), respectively (Figure 5C, 5E, S17). Meanwhile, significantly reduced Treg levels were found in the distal tumors after the combination of PTT with PD-1/PD-L1 checkpoint blockade therapy (Figure 5D and F), suggesting that the combination strategy of PTT with immunotherapy obviously reversed the immune tolerance of the distal tumors. Moreover, the CD8+/Treg ratio was an important parameter to reflect the efficacy of antitumor immune response, which was significantly increased in distal tumor of the mice treated with combined PTT-immunotherapy (Figure 5G).

Based on the analysis of the above data, the following mechanism was proposed. PTT led to the destruction of primary tumor and produced a large number of tumor debris. Subsequently, the tumor associated antigens were released, which could induce an intense immune response in the presence of immune response amplifier IQ, thus effectively recruiting and activating CD8+ and CD4+ T cells into distal tumors. Meanwhile, PD-1/PD-L1 blockade could effectively inhibit the activity of the immunosuppressant Treg, further promote antitumor immunity to suppress the growth of distal tumors.

### Long term immune memory effects

**Figure 6:**
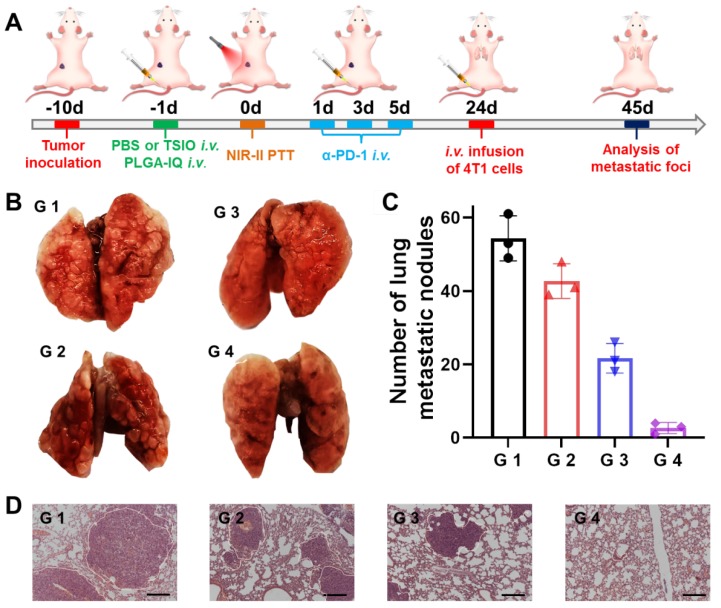
**(A)** Schematic illustration of the experimental design for metastatic neoplasm. **(B)** Representative pictures of lung tissues. **(C)** Quantification of lung metastatic foci and **(D)** H&E staining of lung tissues in different groups (scale bar: 50 *μ*m) (n=3). G1 (PBS +1064 nm laser), G 2 (PBS + 1064 nm laser + *α*-PD-1), G 3 (TSIO + magnet + 1064 nm laser) and G 4 (TSIO + magnet + 1064 nm laser + *α*-PD-1).

Immunological memory effect is one of the basic principles of immune response, which could activate the immune system to protect the body's tissue from previously encountered pathogens. In our study, immunological memory efficacy was evaluated in a whole body diffusion tumor model that was injected intravenously with 4T1 cells on the 24^th^ day after the start of treatments. After another 21 days of feeding mice, different groups of lung tissue were harvested for metastasis analysis (**Figure 6**A). Lung metastatic foci were obvious in all groups, except in the G4 group (Figure 6B and C), suggesting PTT plus *α*-PD-1 therapy could effectively suppress the pulmonary metastasis. Moreover, remarkably less lung metastatic nodules were found in G 4 groups other than in the other three groups (Figure 6D). These results further demonstrated the excellent long term immune memory effect induced by PTT with PD-1/PD-L1 checkpoint blockade therapy.

## Conclusion

We rationally designed the multifunctional TSIO nanoagents that were successfully used to treat primary tumor, distal tumor and metastatic tumor by combining PTT and immunotherapy. The TSIO nanoplatform exhibited enhanced accumulation within the tumor region when a magnetic field was applied and had the ability to real time monitor the treatment process *via* dual NIR-II PA imaging and *T_2_*-weighted MR imaging. In addition, the NIR-II PTT can effectively control and prevent tumor metastasis by stimulating natural and adaptive immunity. The tumor associated antigens released post-PTT, which combined *α*-PD-1 to blockade the PD-1/PD-L1 pathway, could induce intense anticancer immune responses. Moreover, the combination of the NIR-II PTT and the checkpoint blockade therapy had a long term tumor control effect on both the primary and distal tumors, thus this combined treatment approach to enable synergistic systematic therapeutic responses after local PTT. The magnetic targeted NIR-II PA/MR imaging guided PTT provided an effective way to reverse the immunosuppression inside a tumor and to cooperate with immunotherapy to improve therapeutic outcome of the primary, distal and metastatic tumors.

## Methods

### Preparation of TSIO nanoagents

The TSIO nanoagents were prepared through hydrophobic-hydrophobic interaction by a one pot self-assembly technique. Briefly, DSPE-mPEG (3 mg) was added into a suspension of TiS_2_ nanosheets (1 mL, 1 mg·mL^-1^) in chloroform. After stirring for 15 min, a suspension of IO NPs (0.2 mL, 5 mg·mL^-1^) in chloroform was added to the mixture and then stirred for 30 min. Five mL of ultrapure water were then added and stirred until the chloroform had completely evaporated. The product was retrieved by centrifugation and resuspended in phosphate buffered saline (PBS) for further use.

### Establishing magnetic targeted animal model

In order to further understand whether magnetic targeting improved the antitumor treatment, a magnetic targeted animal model was established. Briefly, 4T1 tumor-bearing mice were intravenously injected with 0.2 mL of TSIO nanoagents in PBS (1 mg·mL^-1^), then a magnet with a radius of 1 cm (magnetic field strength = 0.2 T) was fixed to the tumor region so as to mediate the accumulation of TSIO nanoagents within the tumor.

### *In vivo* NIR-II PA imaging

For *in vivo* PA imaging, tumor-bearing mice were injected intravenously with a 0.2 mL suspension of TSIO nanoagents (1 mg·mL^-1^) in PBS prior to imaging. PA images of the tumor site of the mice both with and without magnetic targeting were acquired, and quantitative analysis of PA signal enhancement was conducted at various time points (4, 8, 12 and 24 h post-injection). *In vivo* PA imaging was conducted using a Vevo LAZR-X PA imaging system (Visual-Sonics Co. Ltd, Toronto, Canada) with wavelength = 1270 nm; frequency = 40 MHz; PA gain = 47.0 dB; 2D gain = 28.0 dB; depth = 10.0 mm and width = 12.8 mm.

### *In vivo* MR imaging

For *in vivo* MR imaging, 4TI tumor-bearing mice were intravenously injected with a 0.2 mL suspension of TSIO nanoagents (1 mg·mL^-1^) before imaging. Mice were anesthetized by using isoflurane and fixed to an animal specific body coil for MR imaging data acquisition. *T*_2_-weighted images were obtained on both axial and coronal planes focusing region of interest of tumor. MR images were collected by employing a multi-slice multiecho sequence with the following parameters: repetition time: 2000 ms, echo time: 30 ms, flip angle: 180, matrix size: 256 × 256, field of view: 40 × 40 mm^2^, slices: 16, slice thickness: 1 mm.

### Abscopal effect on bilateral murine 4T1 tumor model

Mice were randomly divided into four groups (n = 5 per group). For the primary tumor inoculation, the 4T1 cells suspended in PBS was subcutaneously injected into the left flank of female BALB/c mouse. For the distal tumor inoculation, which was performed 10 days later, 4T1 cells suspended in PBS was subcutaneously injected into the right flank of female BALB/c mouse. When the primary tumors reached about 60 mm^3^, various treatments were performed.

### Anti-metastasis effect

4T1 cells (1×10^6^) suspended in PBS were subcutaneously infused into the left flank of each female BALB/c mouse. 10 days later, the mice were treated with the approach as same as that of primary tumor model. 24 more days, each mouse was intravenously injected with 5× 10^5^ 4T1 tumor cells. 21 more days, the mice were sacrificed to harvest their spleens for analysis.

## Supplementary Material

Supplementary experimental section and figures.
