# Supplementary material for: Magnetic targeted near-infrared II PA/MR imaging guided photothermal therapy to trigger cancer immunotherapy
Source: Theranostics. 2020 Apr 6;10(11):4997–5010. doi: 10.7150/thno.43604 (PMC7163437; doi:10.7150/thno.43604)
Supplement: Supplementary file 1 — Supplementary experimental section and figures. [file thnov10p4997s1.pdf]

Supporting Information

Magnetic Targeted Near- Infrared II PA/MR Imaging-Guided Photothermal Therapy to Trigger Cancer Immunotherapy

*Qinrui Fu[1#], Zhi Li[1#], Jiamin Ye[1], Zhong Li[1], Fengfu Fu[1], Syue-Liang Lin[2], Cheng Allen Chang[2], Huanghao Yang[1], Jibin Song[1]**

1. *MOE Key Laboratory for Analytical Science of Food Safety and Biology, College of Chemistry, Fuzhou University, Fuzhou 350108, P. R. China.*
2. *Department of Biomedical Imaging and Radiological Sciences, National Yang-Ming University, Taipei 122, Taiwan (ROC)*

[#]Q. Fu and Z. Li contributed equally to this work.

*Corresponding author: jibinsong@fzu.edu.cn

Supplementary Experimental Section

Materials and Reagents**:** All of the chemicals reagents were used without further purification. All reagents were acquired from Aladdin Chemical Reagent other than $Fe(acac)_3$, 1, 2-dodecanediol and benzyl ether, which were purchased from Sigma-Aldrich; 1, 2-distearoyl-sn-glycero-3-phosphoethanolamine-N-[methoxy(polyethylene glycol)-3000 was purchased from sinopeg.com, Xiamen, Fujian Province, P. R. China. ELISA kits (TNF-α, IFN-γ, IL-12) were supplied by Elabscience Biotechnology Co., Ltd. Anti-mouse CD4 monoclonal antibody (FITC conjugated), anti-mouse CD8a monoclonal antibody (PE conjugated or FITC conjugated) and anti-mouse Foxp3 monoclonal antibody (PE conjugated) were supplied from BD biosciences.

Characterization**:** Transmission electronic microscopy (TEM) images were obtained using an HT7700 transmission electron microscope (HITACHI, Japan) at an acceleration voltage of 100 kV. Atomic force microscopy (AFM) images were achieved by a Bruker Dimension Icon (Bruker, USA). Ultraviolet–visible–near-infrared light (UV–*vis*–NIR) absorption spectra were reported using a UH4150 spectrophotometer (HITACHI, Japan). The fluorescence images of cells were achieved by confocal laser scanning microscopy (CLSM, Nikon, Japan). Flow cytometric analysis was obtained using a BD FACS CantoTM ^{II} Flow Cytometer equipped (USA). Photothermal irradiation was performed employing a KS3-11312-110 semiconductor laser unit (BWT, Beijing Kaipulin Co., Ltd., China). An IR thermal camera (FLIR A35, Sweden) was used to acquire photothermal temperature. PA imaging experiments were tested by using the Vevo LAZR-X PA imaging system (Visual-Sonics Co. Ltd, Toronto, Canada). MR imaging experiments were performed on a Bruker 7 T scanner (Pharmascan) equipped with a small

animal-specific body coil.

***Synthesis of TiS_2 Nanosheets*:** The TiS_2 nanosheets were prepared through a modified colloidal chemistry approach. Briefly, titanium tetrachloride (1.1 mL, 10 mmol) was injected into the mixed solvent of octadecen (30 mL) and oleylamine (7.4 mL, 22.4 mmol) under argon protection, heated at 220 °C for 0.5 h; then carbon disulfide (2.35 mL, 40 mmol) was injected into the reaction solution, heated at 300 °C, after 3 h, the reaction was stopped and naturally cool to room temperature. The products were washed and stored in chloroform for further use.

***Synthesis of IO NPs*:** The IO NPs was prepared via the classical thermo-decomposition strategy. Brief, $Fe(acac)_3$ (1412 mg, 4 mmol), oleylamine (4 mL, 12 mmol), oleic acid (3.8 mL, 12 mmol), 1, 2-dodecanediol (810 mg, 20 mmol), and benzyl ether (40 mL) were added into a three-necked flask under argon protection, the reaction solution was heated at 200 °C for 2 h then 300 °C for 1 h. The reaction was stopped and naturally cooled to room temperature, the products were washed and stored in chloroform for further use.

***Cell Culture and Cytotoxicity Assay*:** 4T1 cells were cultured in Dulbecco's modified eagle medium (DMEM, Hyclone) supplemented with 10% fetal bovine serum (FBS) at 37°C within an atmosphere containing 5% CO_2. For the cytotoxicity assays of the TSIO nanoagents, 4T1 cells were seeded in 96-well plates, cultured for 24 h then incubated with various concentrations of a TSIO nanoagents dispersion for 24 h at 37°C. Cell viability was ascertained using a Cell Counting Kit-8 (CCK-8), in accordance with the manufacturer's instructions.

***In vitro PA Imaging*:** *In vitro* PA imaging was achieved using a Vevo LAZR-X PA imaging system (Visual-Sonics Co. Ltd, Toronto, Canada). Briefly, a number of laser wavelengths, including those in the NIR-I (680-970 nm) and NIR-II (1200-2000 nm) windows were employed

to gather PA signal information. In addition, PA signals were collected at optimized excitation irradiation of 1270 nm (NIR-II) at various concentrations of a TSIO nanoagent dispersion (0, 0.075, 0.125, 0.25, 0.50 and 1.0 mg·mL^{-1}).

In vitro MR Imaging: MR imaging and relaxation times of the IO NPs and TSIO nanoagents were obtained using a Bruker 7 T scanner (Pharmascan). Samples of IO NPs and TSIO nanoagents containing 1% agarose gel were fabricated into tubes at the following Fe concentrations: 0.24, 0.12, 0.08, 0.04 and 0.02 mM. MR images were collected by employing a spin echo sequence with the following parameters: repetition time: 2000 ms; echo time: from 10 to 160 ms with a 10 ms interval; matrix: 256 × 256; field of view: 40 × 40 mm^2; slice thickness: 3.00 mm. T_2 relaxation times were obtained by fitting multiple spin echo images.

Establishing Magnetic-Targeted Animal Model: To enhance tumor accumulation of the TSIO assisted by magnetic field, the magnetic-targeted animal model were established. Briefly, 4T1 tumor-bearing mice were intravenously injected with 0.2 mL of TSIO nanoagents in PBS (1 mg·mL^{-1}), then a magnet with a radius of 1 cm (magnetic field strength = 0.2 T) was fixed to the tumor region so as to mediate the accumulation of TSIO nanoagents within the tumor.

PA Imaging of the 4T1 Cell With or Without Magnetic Targeting: The 4TI cell (10^5 cells/dish) were cultured for 24 h and then removing the nutrient solution and washing with PBS, herein, place a magnet at the bottom of one petri dish and the other without a magnet, then the cells were incubated with nutrient solution containing the concentration of TSIO (50 μg·mL^{-1}). After 12 h, the cells were washed and then collected by centrifugation. The obtained cells were dispersed in PBS for PA imaging at 1270 nm laser irradiation.

Flow Cytometry of Annexin V-FITC and PI Co-Staining: 4TI cells were cultured with 50

$\mu g \cdot mL^{-1}$ of TSIO dispersion for 12 h under the applied magnetic field (Figure S6), followed by 1064 nm laser irradiation for 10 min and further incubated for another 4 h. The 4TI cells were collected and stained with PI and Annexin V-FITC immediately before assay by flow cytometry (10,000 cells were counted for each event).

Animal Model: Animal and imaging experiments were performed under a protocol approved by the Fuzhou University Institutional Animal Care and Use Committee. In addition, all experimental animal methods were carried out according to the guide for the care and use of laboratory animals (Ministry of Science and Technology of China, 2006).

The BALB/c mice were each subcutaneously injected with 1×10^6 4TI cells in the left leg or both legs to generate single tumor or double tumor animal model. The tumor-bearing mice were used for imaging and *in vivo* experiments when the tumor volumes reached approximately 50-100 mm^3.

Preparation of PLGA-IQ nanoparticles: The PLGA-IQ nanoparticles were synthetized by a water/oil/water double emulsion approach. Firstly, 0.5 mL 1% w/v PVA solution was employed as water phase, the oil phase was prepared with 2 mL CH_2Cl_2 containing 5 mg/mL PEG-PLGA. To gain the first emulsion solution, the CH_2Cl_2 solution containing 5 mg/mL PEG-PLGA was dropwise added to the PVA solution via sonication under ice water bath. Afterwards, imiquimod dispersed in 5 mg/mL DMSO was mixed in 8 mL 2.5% w/v PVA solution for the second emulsion process. Then, the mixture was homogenized to generate PLGA-IQ nanoparticles. The PLGA-IQ was purified by centrifugation to remove unloaded imiquimod and micronized PEG-PLGA.

Inhibition of Primary Tumor Growth: 4T1 cells (1×10^6) suspended in PBS were subcutaneously

injected into the left flank of each female BALB/c mouse. Mice with a tumor volume of about 60 mm^3 were randomly divided into four groups (n = 5 per group): PBS + 1064 nm laser; PBS + 1064 nm laserv +α-PD-1; TSIO + magnet + 1064 nm laser; and . TSIO + magnet + 1064 nm laser +α-PD-1. PBS or TSIO nanoagents and 1 mg/kg PLGA-IQ were injected intravenously and a an applied magnetic field was employed to promote high tumor accumulation of NPs, 1064 nm laser irradiation for 5 min was used to the mice of different groups on day 1 post injection, subsequently, mice were intravenously injected with α-PD-1 at the dose of 15 μg per mouse on day 1, 3 and 5 post 1064 nm laser radiation. Tumor size were measured using a digital caliper every four days for 24 days. Tumor volume was calculated by $width^2 \times length/2$.

Cytokine detection: Serum were isolated from mice after different treatments and diluted for analysis. Pro-inflammatory cytokines of tumor necrosis factor α (TNF-α), interferon-γ (IFN-γ) and interleukin-12 (IL-12) were analyzed by enzyme-linked immunosorbent assay (ELISA) according to vendors' instructions.

Ex vivo analysis of different groups of T cells: To evaluate the immune cells in distal tumors. The animals were sacrificed 24 days after treatment, and the tumors were harvested. The tissues were minced in Hanks Balanced Salt Solution (HBSS, Hyclone) containing 2% FBS and 2% Ethylenediaminetetraacetic acid (EDTA, 1mM), and filtered through a 40 μm pores size mesh. After repeating the homogenization and filtration steps twice more, the cells were centrifuged at 2000 rpm for 5 min at 4 °C. The cells were counted, and stained with anti-mouse CD4 monoclonal antibody (FITC conjugated), anti-mouse CD8a monoclonal antibody (PE conjugated or FITC conjugated) or anti-mouse Foxp3 monoclonal antibody (PE conjugated) antibodies cocktail in the dark for 30 mins. The stained cells were acquired in the BD FACS $CantoTM^{II}$ flow

cytometer.

Ex vivo fluorescence imaging:Tumor-bearing mice were sacrificed 24 days after treatment, and the distal tumor tissues were harvested, fixed, and labeled with anti-CD8α (D4W2Z) XP® (CD8+ T cells), anti-CD4 (D7D2Z) XP® rabbit monoclonal antibodies for multicolor fluorescence imaging.

***Calculation of the Photothermal Conversion Efficiency*:**

$$\eta = \frac{hs(T_{max}-T_{surr})-Q_{dis}}{I(1-10^{-A_{\lambda}})} \qquad (1)$$

where h represents heat-transfer coefficient, S represents the surface area of the container, T_{max} is the maximum temperature, and T_{Surr} represents the surrounding temperature, Q_{dis} represents the heat dissipated from the photoabsorption of the quartz cuvette sample cell itself. I is the incident energy of the NIR laser (mW), A_{λ} is the absorbance of the TSIO nanosheets at the NIR laser wavelength (λ) of 808 or 1064 nm, and η is the photothermal-conversion efficiency can be calculated according to quation 1.

$$hs = \frac{mc}{\tau_s} \qquad (2)$$

Where m is the mass of water (0.5 g), c is he specific heat capacity of water (4.2 J g^{-1}). The associated time constant of the experimental system, τ_s, can be calculated according to quation 3:

$$\tau_s = -\frac{t}{\ln(\theta)} \qquad (3)$$

Where t is irradiation time, θ is the driving force temperature which is calculated according to equation 4:

$$\theta = \frac{T - T_{surr}}{T_{max} - T_{surr}} \qquad (4)$$

Where T_{max} are the maximum temperature.

Q_{dis} measured independently to be 0.2927 mW, the (T_{max}-T_{surr}) was 34 °C, I is 785 mW, A_{1064} is the absorbance (0.714) of TSIO nanosheets at 1064 nm, hs was measured to be 8.1 mW/°C. Therefore, η calculated to be 45.51% upon laser irradiation using 1064 nm lasers. The η value for 808 nm laser calculated by the same method was 46.82%.

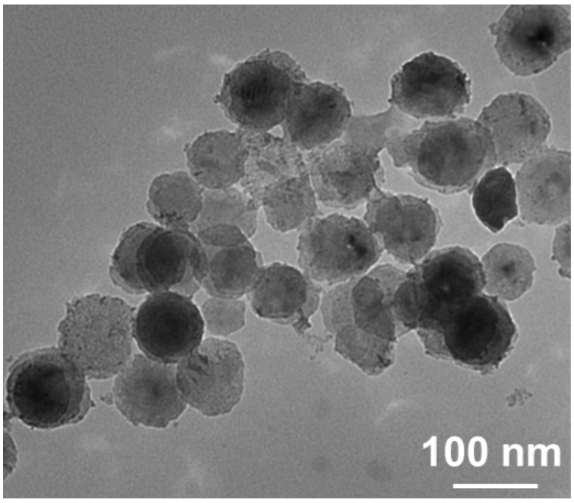

Figure S1. TEM image of DSPE-mPEG-modified TiS_2.

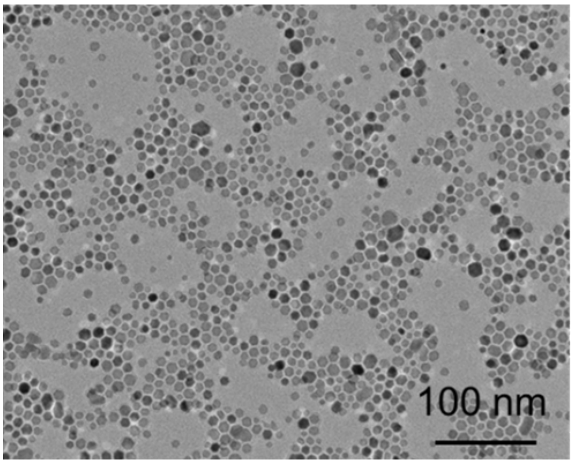

Figure S2. TEM image of magnetic oxide iron (IO) NPs.

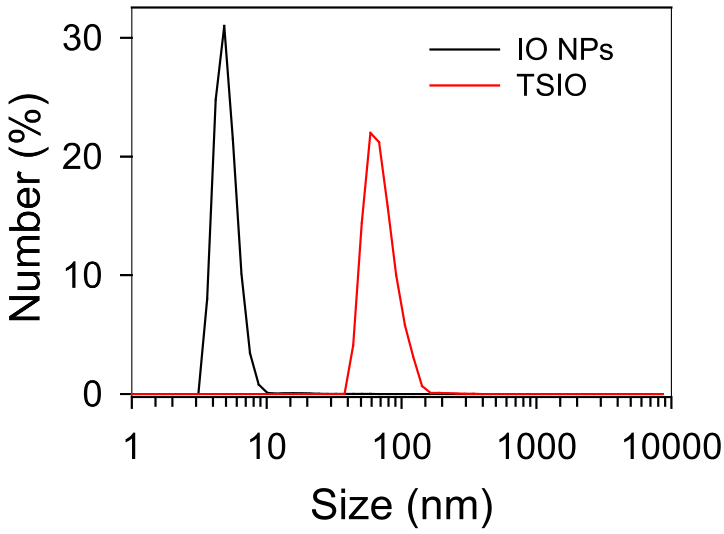

Figure S3. DLS data of IO NPs and TSIO.

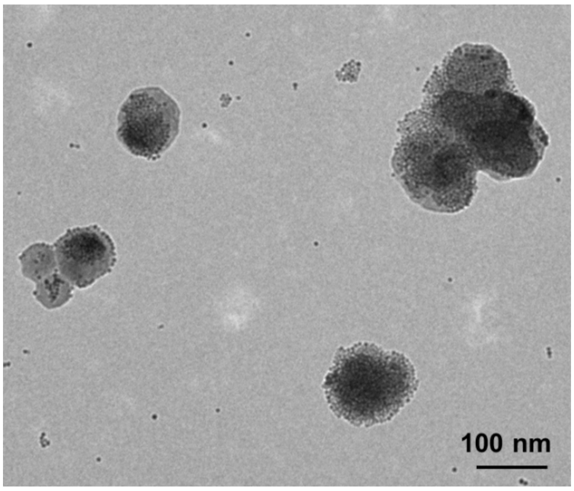

Figure S4. TEM image of nanoagents.

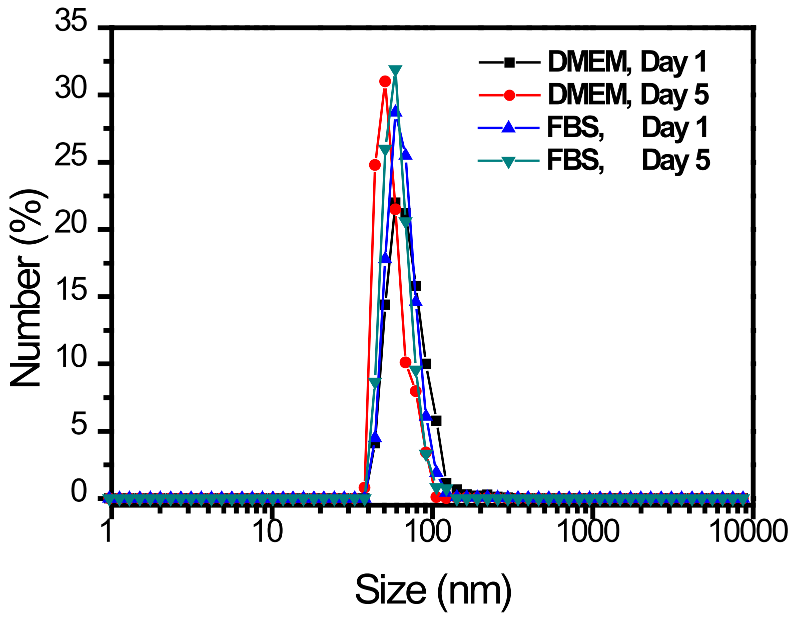

Figure S5. DLS data of the TSIO nanoagents cultured in Dulbecco's modified eagle medium (DMEM) and fetal bovine serum (FBS) for 1 day and 5 days, respectively.

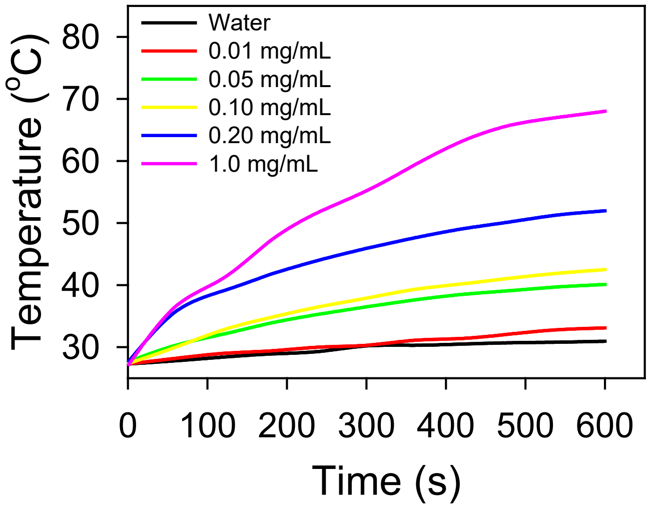

Figure S6. Temperature variation of different concentration of TSIO under 808 nm NIR laser irradiation.

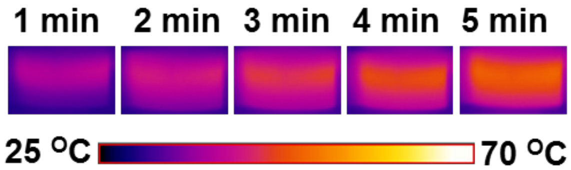

Figure S7. IR thermal images of 1 mL TSIO aqueous solution (1.0 mg·mL^{-1}) using 1064 nm laser irradiation for different time.

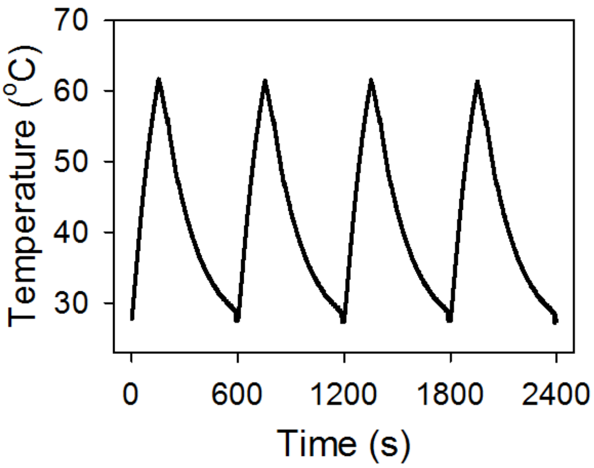

Figure S8. Repeated heating and cooling profiles of aqueous solution of TSIO (1.0 mg·mL^{-1}) under irradiation of 1064 nm laser (1.0 W·cm^{-2}, 5 min).

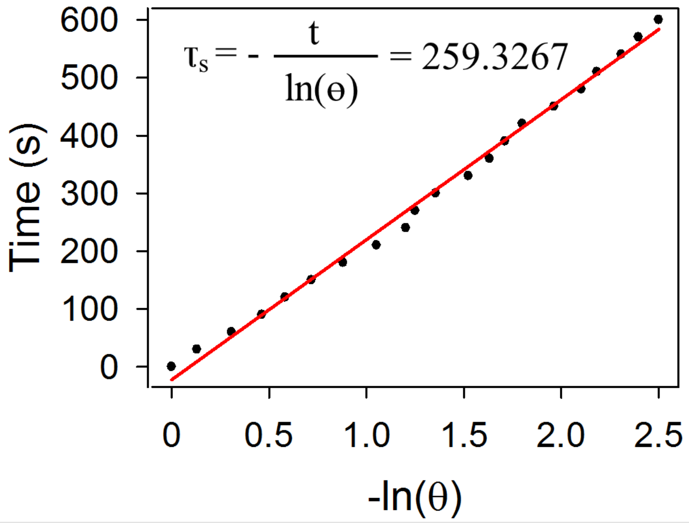

Figure S9. Time constant for heat transfer from the system was determined to be = 259.3267 s by applying the linear time data from the cooling period *versus* negative natural logarithm of driving force temperature, which was obtained from the cooling stage of Figure S8.

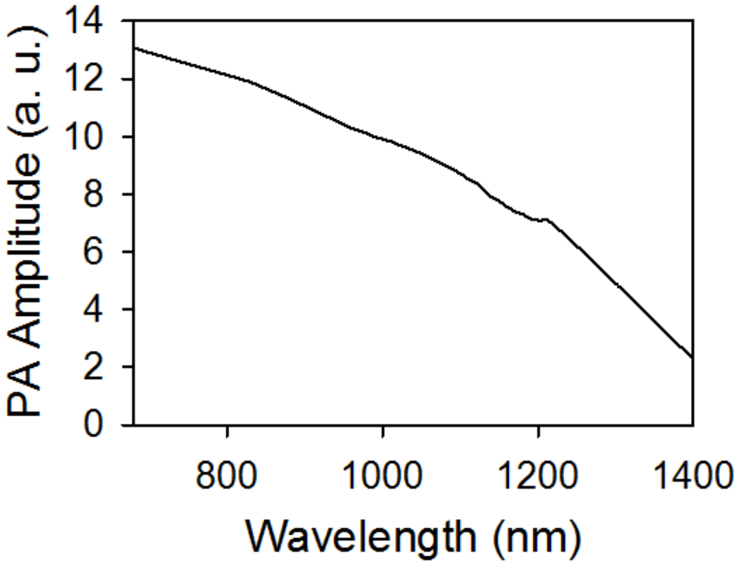

Figure S10. The fitting PA spectrum of TSIO dispersion (1 mg·mL^{-1}).

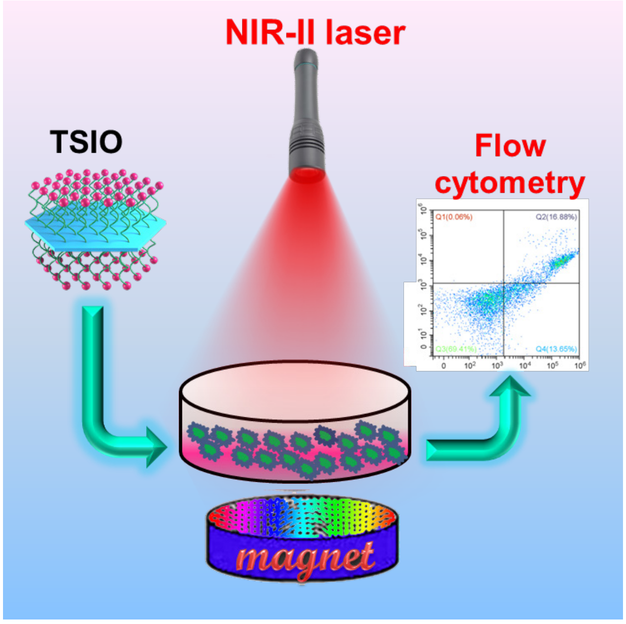

Figure S11. Schematic diagram for magnetic targeting-mediated cell uptake of TSIO using as flow cytometry analysis.

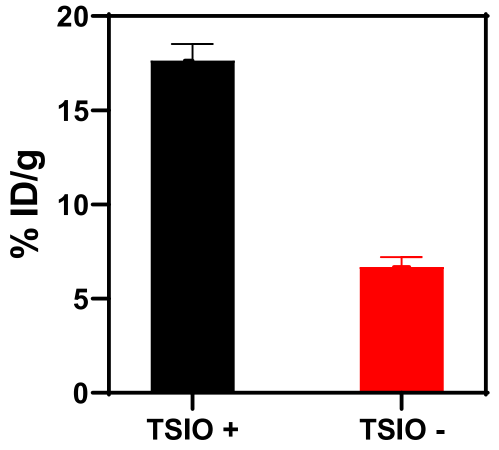

Figure S12. The tumor accumulation efficiency of TSIO nanoagents with (TSIO +) and without (TSIO -) magnetic targeting in mice determined by ICP-MS measured Ti concentrations (24 h post-injection).

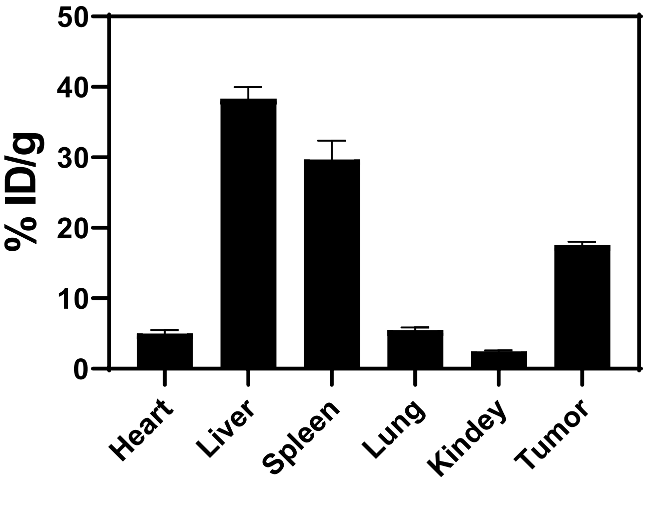

Figure S13. Biodistribution of TSIO in mice determined by ICP-MS measured Fe concentrations (24 h post-injection).

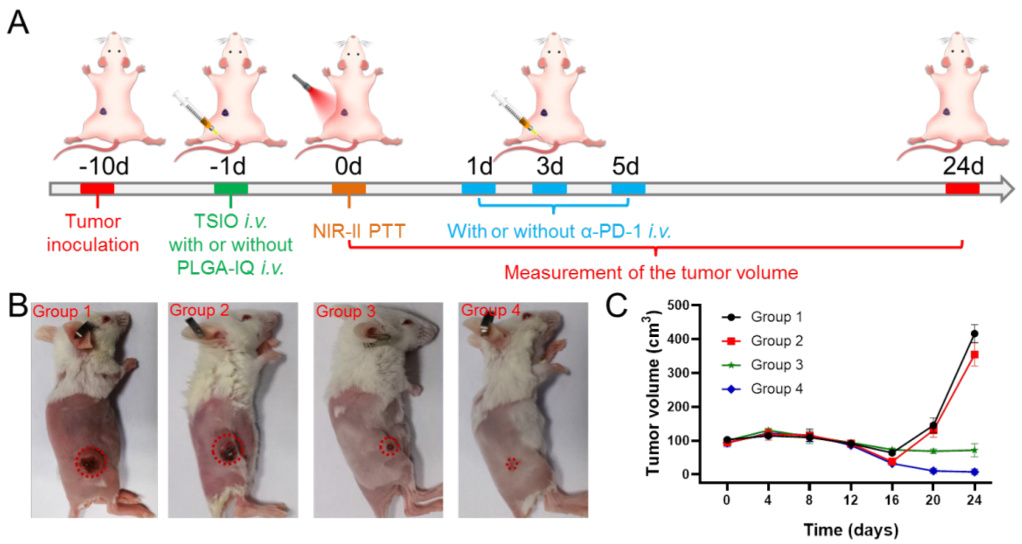

Figure S14. (A) Schematic illustration of supplementary experimental design for primary tumor therapy. Representative photographs (B) and volume (C) of primary tumors in different treatment groups: Group1 (TSIO + NIR-II laser), Group 2 (TSIO + PLGA-IQ + NIR-II laser), Group 3 (TSIO + NIR-II laser + α-PD-1) and Group 4 (TSIO + PLGA-IQ + NIR-II laser + α-PD-1).

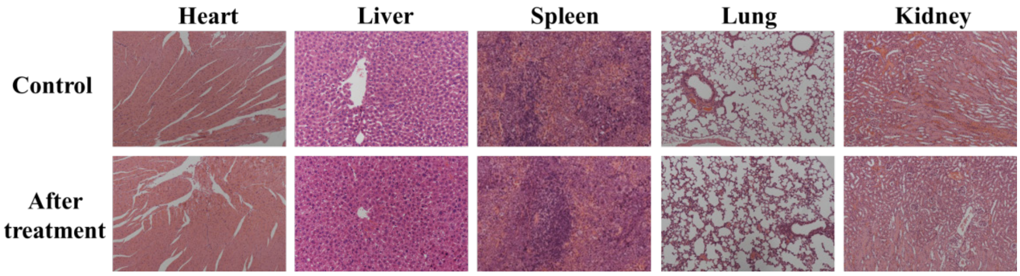

Figure S15. Hematoxylin and eosin stained images of major organs. TSIO injected mice that survived after photothermal-immune combination therapy (with tumors eliminated) were sacrificed 40 days after treatment. Untreated healthy mice were used as the control.

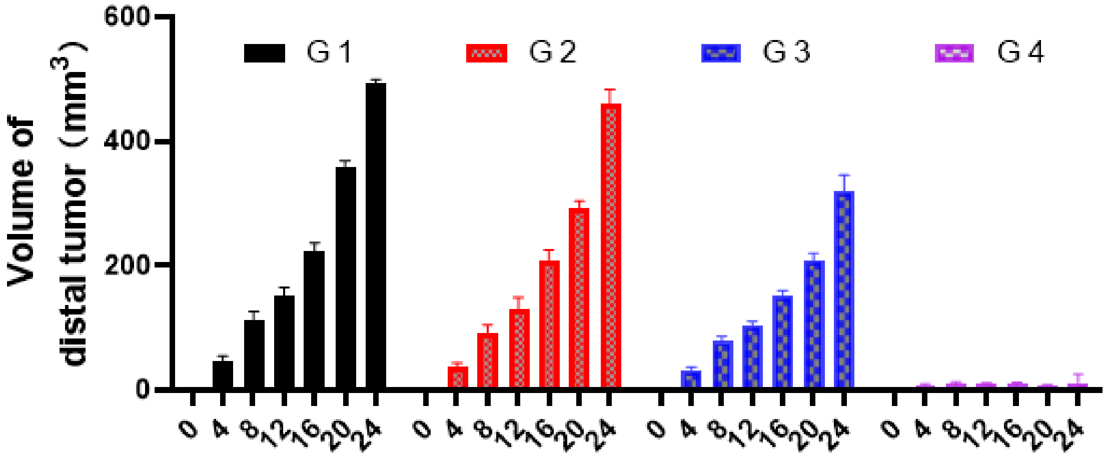

Figure S16. The volume of distal tumors.

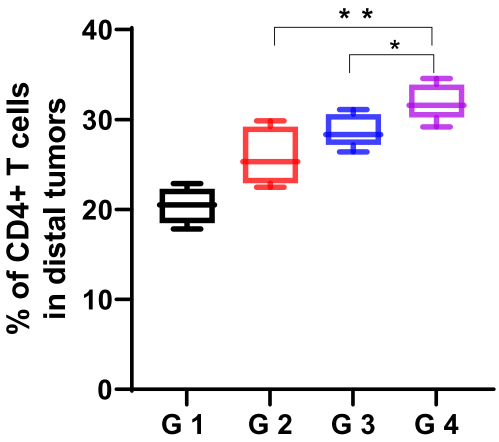

Figure S17. Proportions of the intratumor infiltration of CD4+ T cells in the distal tumors.
